# Feasibility of achieving different protein targets using a hypocaloric high-protein enteral formula in critically ill patients

**DOI:** 10.1186/s13054-021-03625-2

**Published:** 2021-06-11

**Authors:** Pierre Singer, Itai Bendavid, Ilana BenArie, Liran Stadlander, Ilya Kagan

**Affiliations:** Department of General Intensive Care and Institute for Nutrition Research, Rabin Medical Center, Beilinson Hospital, Sackler School of Medicine, Tel Aviv University, Jabotinsky Street, 49100 Petah Tikva, Israel

**Keywords:** Critical illness, Intensive care unit, High protein, Nitrogen excretion, Protein target, Enteral nutrition

## Abstract

**Background and aims:**

Combining energy and protein targets during the acute phase of critical illness is challenging. Energy should be provided progressively to reach targets while avoiding overfeeding and ensuring sufficient protein provision. This prospective observational study evaluated the feasibility of achieving protein targets guided by 24-h urinary nitrogen excretion while avoiding overfeeding when administering a high protein-to-energy ratio enteral nutrition (EN) formula.

**Methods:**

Critically ill adult mechanically ventilated patients with an APACHE II score > 15, SOFA > 4 and without gastrointestinal dysfunction received EN with hypocaloric content for 7 days. Protein need was determined by 24-h urinary nitrogen excretion, up to 1.2 g/kg (Group A, *N* = 10) or up to 1.5 g/kg (Group B, *N* = 22). Variables assessed included nitrogen intake, excretion, balance; resting energy expenditure (REE); phase angle (PhA); gastrointestinal tolerance of EN.

**Results:**

Demographic characteristics of groups were similar. Protein target was achieved using urinary nitrogen excretion measurements. Nitrogen balance worsened in Group A but improved in Group B. Daily protein and calorie intake and balance were significantly increased in Group B compared to Group A. REE was correlated to PhA measurements. Gastric tolerance of EN was good.

**Conclusions:**

Achieving the protein target using urinary nitrogen loss up to 1.5 g/kg/day was feasible in this hypercatabolic population. Reaching a higher protein and calorie target did not induce higher nitrogen excretion and was associated with improved nitrogen balance and a better energy intake without overfeeding. PhA appears to be related to REE and may reflect metabolism level, suggestive of a new phenotype for nutritional status.

Trial registration 0795-18-RMC.

## Introduction

Nutritional therapeutic goals for critically ill patients should be aimed at minimizing the potential for malnutrition while avoiding overfeeding [1]. Timing, route, and energy and protein targets of medical nutritional therapy should be considered equally in developing a comprehensive and individualized approach to nutrition in this population. The European Society of Parenteral and Enteral Nutrition (ESPEN) practice guidelines recommend initiation of enteral nutrition (EN) in the early phase of acute illness, that is, within 48 h of intensive care unit (ICU) admission [1].

Protein catabolism is a common concurrent event of critical illness leading to early and rapid muscle wasting [2]. Because sufficient exogenous protein provision can mitigate skeletal muscle atrophy and improve some clinical outcomes [3, 4], professional guidelines endorse higher than normal daily protein intakes of between 1.2 and 2 g/kg/d [1, 5] in critical illness. These guidelines are, for the most part, based on observational studies due to a lack of evidence from randomized controlled trials. Evidence from a retrospective study indicates significantly lower mortality when greater than 1.2 g/kg/d of protein as compared to less than 1.2 g/kg/d protein is provided to critically ill patients [6]. Achieving nutritional targets with EN in the ICU is difficult due to a myriad of patient-related factors (i.e., co-morbidities, gastrointestinal intolerance, age, body weight) and setting-related factors (i.e., management protocols, staffing numbers and practices, equipment availability) [7].

An estimation of protein requirements is possible using 24-h urinary nitrogen excretion, which is based upon the regression of nitrogen balance on intake. In a randomized trial, for example, 97% of the protein goal of 1.5 g/kg/day was achieved in the intervention group using nitrogen excretion [8]. Nitrogen balance, the difference between nitrogen intake and loss, reflects gain or loss of total body protein and is a reliable measure of dietary protein adequacy [9]. A positive nitrogen balance is indicative of an anabolic state whereas a negative balance indicates a catabolic state. Nitrogen equilibrium in critically ill patients is generally achieved with a nitrogen balance within − 4 g/day or − 5 g/day to + 4 g/day or + 5 g/day [10]. An improved nitrogen balance achieved by increasing protein intake may provide clinical benefits for critically ill patients [11]. Energy requirements evaluated by resting energy expenditure (REE) can be measured using indirect calorimetry in ventilated patients, and is recommended in professional practice guidelines as a means of determining energy requirements in ICU patients [1, 5]. The use of phase angle (PhA) obtained by bioelectrical impedance is considered to be a reliable prognostic parameter for malnutrition [12, 13].

The objective of this exploratory study was to investigate the feasibility of progressively achieving protein targets, as defined by 24-h urinary nitrogen excretion, while avoiding exceeding energy targets guided by indirect calorimetry when administering a high-protein EN formula to critically ill patients.

## Materials and methods

### Patients

This unblinded, single-center observational two-phase study evaluated the feasibility of achieving protein targets as prescribed by results of 24-h urinary nitrogen excretion. The aim of nutritional therapy was to meet protein targets but not exceed energy requirements.

The intention was to include a convenience sample of 32 patients admitted to the intensive care unit of a hospital in Israel. Eligible for the study were acutely ill patients who were expected to be on mechanical ventilation and to remain in the ICU for at least 48 h, were older than 18 years and who either provided signed informed consent themselves or a legal representative provided consent. Further enrollment criteria were an acute physiology and chronic health evaluation (APACHE II) score > 15 and a Sequential Organ Failure Assessment (SOFA) score > 4. Patients showing signs of gastrointestinal (GI) dysfunction or failure, uncontrolled shock, uncontrolled hypoxemia or acidosis, liver failure or acute kidney injury, with burn injuries, documented clostridium difficile infection, a body mass index (BMI) > 40 kg/m^2^, galactosemia and/or a congenital inability to metabolize nutrients were not eligible for study inclusion.

Approval of the study protocol was obtained from the Rabin Medical Center Institutional Review Board.

### Study nutrition

Study nutrition was started enterally via nasogastric tube on the day of ICU admission and was provided for at least 5 days for a maximum of 7 days or until the initiation of oral intake, discharge from the ICU or patient death. We used an EN formula (Fresubin Intensive®, Fresenius-Kabi, Bad Homburg, Germany) containing 12.9 g/100 mL carbohydrate (42% of calories), 3.2 g/100 mL fat (24% of calories), 10 g/100 mL protein (33% of calories), 0.64 g/100 mL fiber (1% of calories). The protein target was determined by 24-h urinary nitrogen excretion, started on the day of ICU admission and the energy target by REE.

Because while receiving protein at the protocol-defined upper limit of 1.2 g/kg/d the target goal of ≥ 80% of REE was not achieved with the first 10 patients enrolled in the study (Group A), it was decided to increase protein to an upper limit of 1.5 g/kg/d in the subsequent 22 patients to achieve this energy target (Group B). Hence, nutrition requirements for Group A were guided by 24-h nitrogen excretion for the duration of the 7-day study. Nutrition requirements in Group B were guided by nitrogen excretion then by a protein target of ≤ 1.5 g/kg/d and an energy target set at ≥ 80% of measured REE from day 5. Rates of EN administration in both groups were adjusted accordingly each day.

### Measurements

Samples of 24-h urinary output were collected daily and analyzed to obtain 24-h urinary nitrogen (from urea) excretion. The calculation of protein intake from nitrogen excretion was based on the following equation: 1 g nitrogen is in 2 g of urea or 6.25 g protein + 4 g of insensible loss. Progression was performed according to the nitrogen value on day 1 and subsequent measurements. EN was administered as a continuous infusion based on the prescription of the protein related to urinary nitrogen excretion and nitrogen insensible losses (4 g).

Weight, height and BMI were recorded daily, SOFA scores were calculated daily. Standard laboratory serum chemistry and hematology tests were obtained; fluid balance and other standard measurements for assessment of patient condition were performed according to local practice guidelines. Bioimpedance for resistance and reactance measurement, body composition (fat mass and fat-free mass) and PhA measurement were performed on day of ICU admission and daily therafter using Quadscan 4000 (Bodystat, UK). REE was calculated from VCO_2_ and VO_2_ measured once daily using indirect calorimetry (Cosmed Q-NRG, Italy). Substrate utilization was calculated according to the equation from Frayn [12].

Safety assessments were performed routinely and comprised the recording of types and severity of adverse events: vomiting; diarrhea, defined as > 3 episodes of liquid stool in 24 h; constipation; gastrointestinal bleeding; tolerance to enteral feeding evaluated by gastro residual volume. Reasons for study or EN withdrawal were recorded.

### Statistical analysis

Due to the pilot character of this study, the sample size is primarily driven by feasibility reasons rather than by a formal sample size calculation. A sample size of 30 was considered realistic in terms of known rates of ICU admissions at the study hospital. The confidence interval for protein balance (i.e., difference between protein intake and the protein loss) was calculated as mean ± *z*(1 − *α*/2) × SD/√*n*, where *z*(1 − *α*/2) equals 1.96 for a significance level *α* = 0.05, where SD is the standard deviation of the protein balance and n the sample size. We used the two-way ANOVA test for repeated measures and Chi test to compare episodes of diarrhea.

Statistical analyses were descriptive in nature given the exploratory character of the study. That is, patient characteristics and nutritional variables are presented as counts and percentages, and means and SD. A comparison between variables of interest was summarized using the point estimate of the difference between means of prescribed, administered and utilized protein and 95% confidence interval. Missing data were not imputed. Data were evaluated as observed. Correlation between REE and PhA measurements as well as between REE and nitrogen excretion were performed using Pearson test analysis.

## Results

Thirty-two patients were included in the study: 10 in Group A and 22 in Group B. The two groups were comparable for most baseline characteristics (Table 1). Patients in Group A were non significantly older than those in Group B, and APACHE II scores were non significantly lower in Group A than in Group B at enrollment.

**Table 1:** Baseline Characteristics of Patients receiving Study Nutrition for 7 Days

	Group A (*N* = 10)	Group B (*N* = 22)
Age, mean, years	68.4 ± 15.2	61.3 ± 15.4
Male sex, number	6 (60%)	13 (59%)
Actual mean body weight, kg	71.4 ± 19.1	74.7 ± 18.8
BMI, kg/m^2^	26.2 ± 6.8	26.4 ± 4.9
Diagnosis on ICU admission, number		
Multiple trauma	2 (20%)	5 (22.7%)
Sepsis	2 (20%)	5 (22.7%)
Respiratory distress/failure	5 (50%)	9 (40.9%)
Other	1 (10%)	3 (13.6%)
APACHE II score day 1, mean	21.7 ± 4.0	24.4 ± 4.6
SOFA score day 1, mean	8.1 ± 2.4	8 ± 3.3
SOFA score day 7, mean	7 ± 2.4	6.9 ± 3.9
Creatinine at ICU admission (mg/dL)	1.7 ± 1.5	1.7 ± 1.4
BUN at ICU admission (mg/dL)	91.8 ± 65.1	101.9 ± 64.6

BMI, body mass index; ICU, intensive care unit; SOFA, Sequential Organ Failure Assessment; BUN, blood urea nitrogen

### Protein intake and nitrogen parameters

**Fig. 1:**
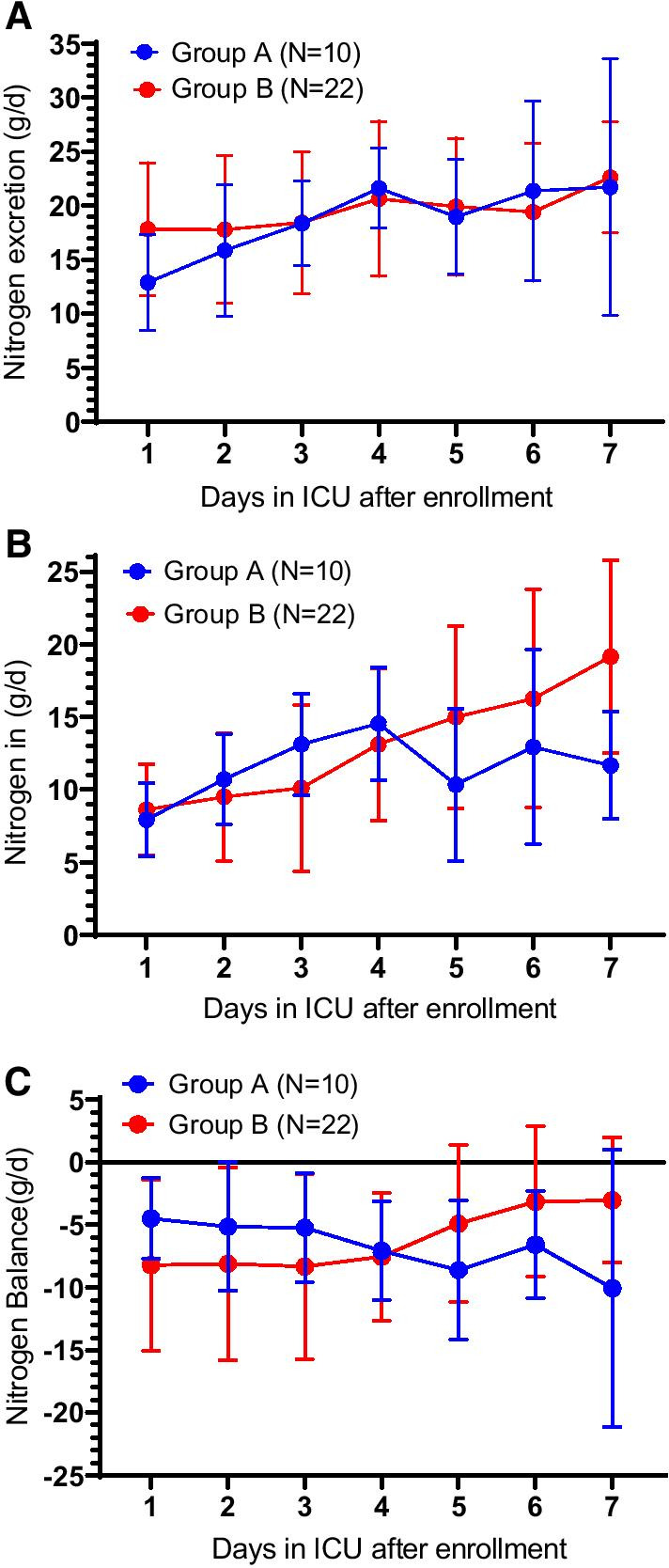
Nitrogen measurements in Groups A and B over the course of the 7-day study. **a** nitrogen excretion, **b** nitrogen intake, and **c** nitrogen balance. Error bars are SD for means in the two groups at each time point. Nutrition requirements for Group A were guided by 24-h nitrogen excretion for the duration of the 7-day study using an upper limit of 1.2 g/kg/d for protein intake. Nutrition requirements in Group B were guided by nitrogen excretion then by an upper limit of 1.5 g/kg/d for protein intake combined with REE from day 5

Patients in both groups were hypercatabolic (Fig. 1) reaching a nitrogen excretion above 18 g in both groups. In Group A, mean nitrogen excretion over 7 days was 18.7 ± 3.3 g/24 h. For the same time period, nitrogen excretion was 19.5 ± 1.6 g/24 h in Group B (no significant difference between groups) (Fig. 1a). Nitrogen intake in Group A increased from 7.9 ± 2.6 g/d on day 1 to 11.6 ± 3.7 g/d on day 7. In Group B, mean nitrogen intake was 8.6 ± 3.1 g/d on day 1 with a two-fold increase to 19.1 ± 6.6 g/d on day 7 (*p* < 0.002) (Fig. 1b).

Nitrogen balance in Group A decreased steadily over 7 days despite a relatively constant protein intake. At day 1, mean balance was − 4.5 ± 3.2 g/d decreasing to − 10.1 ± 11.1 g/d at day 7. By contrast, nitrogen balance in Group B steadily increased from − 8.2 ± 6.8 g/d at day 1 to ± − 3 5 g/d at day 7 (*p* < 0.03) (Fig. 1c).

A comparison of protein intake and nitrogen parameters shows that while protein intake remained relatively unchanged in Group A, nitrogen excretion increased and nitrogen balance worsened. In comparison, as protein intake increased in Group B, nitrogen excretion increased with an improvement in nitrogen balance (Fig. 1a, c, Table 2). These changes were significant between groups (nitrogen intake *p* < 0.002 and nitrogen balance *p* < 0.03).

**Table 2:** Comparisons of Energy and Protein Intake, Resting Energy Expenditure and Phase Angle Measurements on Days 1, 3, 5 and 7 in Groups A and B

	Group A *N* = 10	n*	Group B *N* = 22	n*
Day 1				
REE, kcal/d	1530.9 ± 460.4	10	1724.4 ± 303.3	20
Total enteral calories, kcal/d	600 ± 195.2	10	688.6 ± 247.4	22
Administered REE	39%		40%	
Protein administered, g/kg/d	0.74 ± 0.3	10	1.03 ± 1.4	22
Calorie balance, kcal/d	− 785.2 ± 421.6	10	− 866.2 ± 354.2	20
Phase angle, degree	3.8 ± 1.3	10	4.5 ± 2.1	18
Day 3				
REE, kcal/d	1626.4 ± 365	10	1668.9 ± 347.7	17
Total enteral calories, kcal/d	992.3 ± 265.3	10	770.5 ± 436.3	19
Administered REE	61%		46%	
Calorie balance, kcal/d	− 2073.4 ± 1207.4	10	− 2441.1 ± 931.4	17
Protein administered, g/kg/d	1.2 ± 0.32	10	0.85 ± 0.53	19
Phase angle, degree	3.3 ± 1.3	8	3.3 ± 1.1	7
Day 5				
REE, kcal/d	1611.3 ± 353.8	10	17,161. ± 334.2	15
Total enteral calories, kcal/d	819.7 ± 374.3	10	1132.4 ± 497.1	16
Administered REE	51%		64%	
Calorie balance, kcal/d	− 3147.9 ± 1801.4	10	− 3135.5 ± 1693.3	15
Protein administered, g/kg/d	0.97 ± 0.52	10	1.2 ± 0.4	16
Phase angle, degree	5.9 ± 3.8	2	3.6 ± 1.4	6
Day 7				
REE, kcal/d	1677.3 ± 563.8	7	1728.9 ± 317.6	11
Total enteral calories, kcal/d	952.6 ± 339.8	7	1457.4 ± 501.7	11
Administered REE	57%		84%	
Calorie balance, kcal/d	− 3943.4 ± 2667	7	− 3382.9 ± 2271.7	11
Protein administered, g/kg/d	1.2 ± 0.39	7	1.4 ± 0.58	12
Phase angle, degree	3.4 ± 1.5	6	5.1 ± 3.9	9

*Patients with available data. Mean ± SD for all values. Administered REE represents percent of resting energy requirements met by EN. Calorie balance represents all intake including intravenous dextrose preparations. REE, resting energy expenditure

### Energy intake

**Fig. 2:**
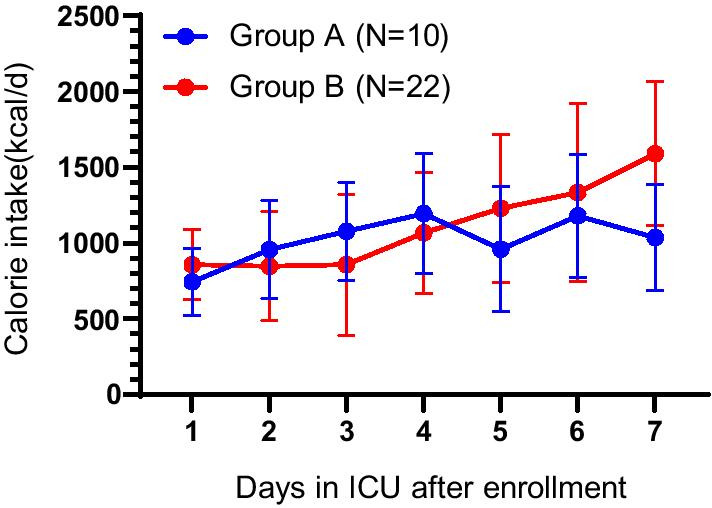
Total calorie intake per day from enteral nutrition in Group A and Group B. Error bars are SD for means in the two groups at each time point

Nutrition therapy for Group A was guided by 24-h nitrogen excretion with a maximum protein intake of 1.2 g/kg/d throughout the 7-day study, whereas EN in Group B was guided by nitrogen excretion then by a maximum protein intake of 1.5 g/kg/d and REE from day 5 on. Energy intake was derived from the protein prescription. In Group A, the mean total calories per day fluctuated and energy intake at day 7 was low at 952 ± 340 kcal or not more than 57% of the measured energy expenditure (Table 2). Mean total calories in Group B increased continually during the study reaching 57% of the measured energy expenditure at day 5 and 84% of the measured REE (1457 ± 501 kcal) on day 7 (*p* < 0.004) (Fig. 2). Differences in energy intake between the two groups were significant (*p* < 0.02).

A comparison of REE and energy intake shows that energy intake was not greater than REE in either group on any day of the study (Table 2). Hence, a negative calorie balance was observed in both groups, and this negative balance increased in both groups over time.

### Correlations

**Fig. 3:**
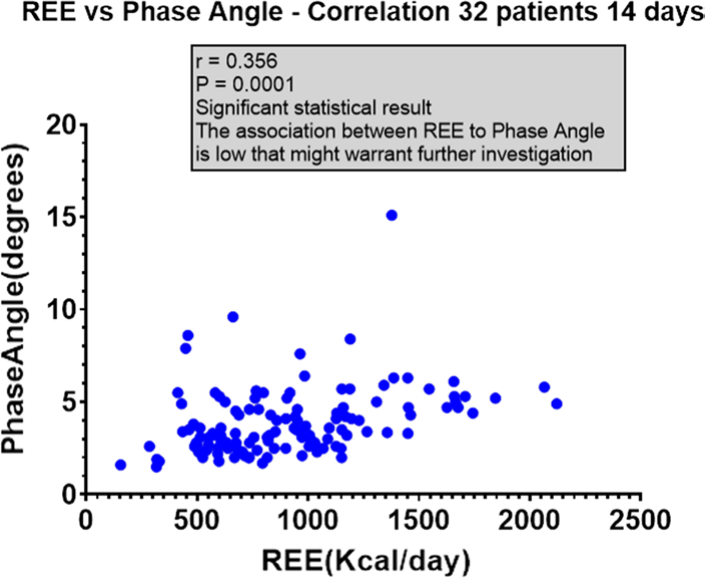
Comparison of REE and phase angle for all 32 patients

Phase angle measurements decreased slightly from day 1 to day 7 in Group A, but showed a slight increase during the study period in Group B (disregarding values from day 5 due to the low number of measurements obtained on that day in each group). There was a strong correlation between REE and phase angle (*r* = 0.356; *p* < 0.0001; 95% CI 0.1917–0.5008) (Fig. 3). Measurements of VO_2_, VCO_2_ and calculations of REE were not significant from day to day within each group or between groups. RQ was significantly lower in Group B (*p* < 0.004). Calculation of carbohydrate and fat utilization were comparable in the two groups.

### Safety

The mean maximum daily residual volume was lower in Group A than Group B at 70.2 ± 74.4 ml/day vs .109.6 ± 140.7 ml/day, respectively, showing a good gastric tolerance to the EN formula. Diarrhea, defined as > 3 episodes in 24-h, occurred in patients in both groups. In total, 11 patients experienced diarrhea (34.4% of 32), five in Group A and six in Group B. Two patients in Group A discontinued the study early due to hypernatremia. There were 3 early withdraws in Group B due to hypernatremia and diarrhea (1 patient), diarrhea (1 patient) and diarrhea and vomiting (1 patient). There was no significant difference in diarrhea episodes between the two groups.

## Discussion

Our study shows that a formula containing a high protein content but with moderate energy load can provide a sufficient protein intake while preserving nitrogen balance and preventing overfeeding in critically ill, mechanically ventilated, hypercatabolic patients during the acute phase of illness (the first 7 days). The acute phase of severe illness is composed of an early period and a late period [1]. The good gastric tolerance of the formula provides an additional argument for its use when striving to meet professional guideline recommendations for protein intake at 1.2–2.0 g/kg/day according to ASPEN [5] and progressive administration of protein at 1.3 g/kg/day as per ESPEN [1]. Providing required energy while progressively meeting protein targets in critically ill patients represents a challenging situation as not all EN formulas provide sufficient quantities of protein to reach protein targets without overfeeding patients. In our study, group A received a hypocaloric regimen during the 7 days of the observational study. Group B did not reach the recommended target recommended by ESPEN (70% of the measured energy expenditure) at day 3 and only 57% of the measured energy expenditure at day 5 and 84% of the measured REE (1457 ± 501 kcal) on day 7. This achievement may be more beneficial than overfeeding obtained by standard enteral formulas less enriched in protein.

Another approach in this situation would be to match the type of formula to the phase of the critical illness: that is, a formula for the acute phase and another for the post-acute phase.

Elwyn [10] demonstrated that nitrogen excretion in an injured population was about 18 g/d, which is comparable to our population. In an earlier review, Elwyn [15] discusses the effect of various levels of energy and protein intake on nitrogen balance. According to data from his work with postoperative and depleted patients, the addition of protein to a low energy intake (below 15 kcal/kg) leads to an improvement in nitrogen balance in a slope of 7.5 mg nitrogen/kcal. However, at energy intake above 15 kcal/kg, the slope is reduced to 1.5 mg nitrogen/kcal [16, 17].

The goal of medical nutrition is to provide enough protein to have a deposition of protein in body cell mass but not a fat deposition in muscle. In our study, we did not find a significant difference in body composition as measured by PhA after 7 days of EN. Group B received more protein and more energy reaching a stable nitrogen balance and an improved energy balance, avoiding overfeeding. Substrate endogenous production occurs in the early period of acute phase illness, providing most of the required substrates [18]. Any additional provision of nutrients may induce carbohydrate load, hyperglycemia, hypertriglyceridemia, increase production of CO_2_ and PaCO_2_, and lipogenesis. A formula that combines high protein content with lower energy intake may provide the required nutritional support required by critically ill patients at the early phases of illness.

Professional organizations [1, 5] and several clinical studies [19–22] recommend a high protein intake to improve outcomes of critically ill patients. In our study, we noted a worsening of nitrogen balance accompanied by an increase in nitrogen in Group A despite a seemingly steady protein intake just meeting the target of 1.2 g/kg/d. By contrast, although nitrogen excretion continued to increase in Group B, we observed improvement in nitrogen balance, which remained below equilibrium but in line with generally accepted ranges [7], with a protein intake below target. The combined effect of improved protein and energy intake may explain this phenomenon. Previous investigations of the association of protein intake with nitrogen loss in critically ill patients seem to suggest that increased protein intake might improve nitrogen balance [23, 24] although the effects of higher protein are not always sustained [20] or are associated with an increase in urinary urea excretion [7]. Results of an observational study indicated a significant improvement in nitrogen balance with a protein intake of 1.5 g/kg/day vs a protein intake of 1.1 g/kg/day [22], a result similar to what we found in the two study groups. Nitrogen balance improved over 7 days in critically ill patients randomized to a protein-fortified diet versus those on a standard diet; differences between groups were significant [25].

We acknowledge that individual variability in lean body mass and the effects of insensible nitrogen loss may have influenced our results on nitrogen balance. Urinary nitrogen loss is the direct reflection of muscle destruction secondary to ubiquitinisation [26]. This process is not easy to attenuate or even to stop despite large amounts of protein administered. Wandraq [27] showed a muscle loss of more than 25% in 14 days despite the administration of 1.2 g/kg/d of protein. Nitrogen balance remained negative at − 7 g/day. Even the Danielis study [25] shows the same negative nitrogen balance after 7 days with a protein intake of 1.8 g/kg/d, suggesting that nitrogen balance could not be the best parameter to follow to evaluate the efficacy of protein intake [28].

An intention of this study was to evaluate in a real practice setting the intake of EN based on individual patient need as opposed to a formula-based standard care in a typical ICU setting. The study showed that protein provision according to individual 24-h urinary N excretion was feasible. The rate of increasing protein administration may have been faster, reaching the target within 3 days without exceeding the energy intake. By changing the target goal from one based on nitrogen excretion to one based on REE, we were able to increase energy intake and slightly increase protein intake without overfeeding patients, as was shown in Group B. The results in the first group of patients (Group A) after using an upper limit of protein intake of 1.2 g/kg/d for 7 days demonstrated the challenge of attaining energy requirements while limiting protein intake.

Protein intake in the critical care setting appears to be the most important macronutrient to support immune function and maintain lean body mass [5] and goals for protein delivery should be reached as early as possible in the ICU stay [29]. For most critically ill patients, protein requirements are proportionately higher than energy requirements [5] although traditionally, an energy rather than a protein target has been used as a strategy to prevent overfeeding. Results from a 2016 RCT [21] combined with those from a previous trial by the same investigators [30], suggest that high protein intake may be a fundamental target independent of caloric delivery in critically ill patients. The EN formula used in this study is a modified formulation with a higher protein to energy ratio, which more appropriately matches requirements in the early acute phase. Most standard enteral formulas do not contain sufficient protein to adequately meet needs during the acute phase without overfeeding calories, especially if the patient is hypercatabolic.

It may be beneficial, therefore, to limit protein debt even more than energy debt. Since the metabolic response to stress is modifying the metabolic needs [19], it is suggested to modify the nutritional regimen accordingly. In this study, the same formula was used throughout the 7 days of the study, but it may be interesting to propose the study formula in the acute phase (early period) and another formula, with a different protein content, toward the end of the late period of the acute phase.

Indirect calorimetry is the recommended method to determine energy expenditure and subsequent calorie target [31]. A major benefit of indirect calorimetry in clinical practice is the prevention of both under- and over-feeding in patients with different conditions because it allows an individualized approach to providing nutrition support and monitoring its effects [32]. Optimal energy delivery targeting REE measured by indirect calorimetry seems to be significantly associated with reduced mortality in ICU patients as found in a retrospective study [33].

PhA seems to be an appropriate prognostic tool for evaluating nutritional status. PhA was extensively measured in this study, even if the main objective of the study was N balance. Improvements in nutrition status, as measured by achieved energy and protein intake goals, were better met when PhA was implemented to control energy needs compared to a formula-based estimate in mechanically ventilated patients [34]. An association between independent PhA scores [35] or PhA considered in multivariate analysis [36] as a possible prognostic marker of outcome in ICU patients has been reported. The results of this study show that PhA improved in Group B, possibly a reflection of improved cellular health and function in these patients. We also observed a relationship between PhA and REE. An explanation for this finding might be that REE usually increases in patients with increased lean body mass, thereby supporting the value of PhA as a strong nutritional parameter and phenotype of muscle mass. However, PhA is also an indicator of inflammation/severity of illness. The improvement observed in the C group may be related to in a decrease in the severity of illness and not to a nutritional improvement.

Approximately one-third of all patients experienced at least one episode of diarrhea. Despite higher daily intakes of EN in Group B, fewer patients in this group experienced diarrhea. Diarrhea is common in critically ill patients with a prevalence estimated to be between 15 and 38% [28]. EN was not identified as an independent risk factor for diarrhea in a randomized study [4]. Diarrhea is often multifactorial and incidence is known to increase as the duration of hospitalization increases.

### Study limitations

The major limitation of this study was the low number of patients and the number of patients who discontinued the study. In addition, in the lack of clear guidelines, a clear strategy for protein intake remains open and our study provides some additional light on this topic.

## Conclusions

Using the study formula and targeting protein administration using N excretion is feasible, safe and improves calorie balance without increasing nitrogen excretion or a negative nitrogen balance. Our results suggest a relationship between PhA and REE and may reflect metabolism level, suggestive of a new phenotype for nutritional status. The use of this formula in the acute phase (days 1 to 5–7) is in line with guidelines for nutritional support in intensive care patients as provided by ESPEN [1]. Large multicenter clinical trials are called for to evaluate protein requirements and that correlate protein intake with clinical outcomes.

## Data Availability

Not applicable.
